# Stimulus Control of Odorant Concentration: Pilot Study of Generalization and Discrimination of Odor Concentration in Canines

**DOI:** 10.3390/ani11020326

**Published:** 2021-01-28

**Authors:** Mallory T. DeChant, Paul C. Bunker, Nathaniel J. Hall

**Affiliations:** 1 Department of Food and Animal Sciences, Texas Tech University, Lubbock, TX 79409, USA; mallory.dechant@ttu.edu; 2 Chiron K9, San Antonio, TX 78251, USA; paul@chiron-k9.com

**Keywords:** canine detection, odor discrimination, concentration generalization

## Abstract

**Simple Summary:**

Dogs are deployed worldwide for detection tasks, but little is known about how they spontaneously generalize between concentration variations of their trained odor. This study found that dogs spontaneously generalized within a 10-fold concentration range lower than the training stimulus. Further, dogs could be trained to discriminate between concentrations within that 10-fold range. However, discrimination training did not affect dogs’ spontaneous generalization to the odor concentration unless discrimination training occurred in compound with generalization testing, suggesting that relative stimulus control of the target and non-target concentrations might be important in determining whether dogs will respond.

**Abstract:**

Despite dogs’ widespread use as detection systems, little is known about how dogs generalize to variations of an odorant’s concentration. Further, it is unclear whether dogs can be trained to discriminate between similar concentration variations of an odorant. Four dogs were trained to an odorant (0.01 air dilution of isoamyl acetate) in an air-dilution olfactometer, and we assessed spontaneous generalization to a range of concentrations lower than the training stimulus (Generalization Test 1). Dogs generalized to odors within a 10-fold range of the training odorant. Next, we conducted discrimination training to suppress responses to concentrations lower than a concentration dogs showed initial responding towards in Generalization Test 1 (0.0025 air dilution). Dogs successfully discriminated between 0.0025 and 0.01, exceeding 90% accuracy. However, when a second generalization test was conducted (Generalization Test 2), responding at the 0.0025 concentration immediately recovered and was no different than in Generalization Test 1. Dogs were then tested in another generalization test (Compound Discrimination and Generalization) in which generalization probes were embedded within discrimination trials, and dogs showed suppression of responding to the 0.0025 concentration and lower concentrations in this preparation. These data suggest dogs show limited spontaneous generalization across odor concentration and that dogs can be trained to discriminate between similar concentrations of the same odorant. Stimulus control, however, may depend on the negative stimulus, suggesting olfactory concentration generalization may depend on relative stimulus control. These results highlight the importance of considering odor concentration as a dimension for generalization in canine olfactory research.

## 1. Introduction

The highly developed olfactory system of dogs has been utilized in the applications of explosives detection [1,2,3,4], narcotics detection [5,6], cancer screening and detection [7,8,9,10] and conservation related detection [11,12,13]. This widespread use of canines for a variety of industries and agencies highlights the need for basic research understanding canine olfactory perception and how to optimize canine training for detection of highly specific targets.

Olfactory generalization, or making the same behavioral response to variations of a learned odor [14], is a key behavioral phenomenon in need of further research for a variety of detection dog applications. For example, dogs trained to one type of oxidizer common in improvised explosive devices (e.g., ammonium nitrate) also need to be able to respond to chemically related oxidizers (e.g., calcium ammonium nitrate) or the same oxidizer when mixed with other materials; however, dogs often fail to spontaneously generalize across these odor variations [3,14,15].

Predicting the degree or magnitude of generalization to different olfactory stimuli can be challenging, because predicting odor “similarity” from chemical structure is not always successful. In non-olfactory domains, generalization across a single stimulus dimension (e.g., hertz for tone or wavelength for light) shows a predictable generalization curve [16,17] in which the highest levels of responding occur to the trained stimulus with systematically less responding to increasingly dissimilar stimuli (of higher or lower values) following a Gaussian-like curve. Such generalization curves have rarely been described or tested for olfactory stimuli, except for a few notable exceptions. Generalization across odorants with the same functional group but vary in the length of the carbon chain follows a predictable generalization gradient [18,19,20,21,22]. In addition, honey bees show a predictable pattern of generalization from a trained binary odor mixture to systematically varying ratios of the binary odor mixture [23].

Interestingly, little work has investigated odor concentration as a dimension for generalization. Perceptual constancy across odor concentration is important for animals to track and locate natural odorants at varying distances from an odor source [24], although little research has evaluated animals’ spontaneous recognition of odors across varying concentrations. In non-chemosensory stimuli, research has previously shown that changes in stimulus intensity produce asymmetric generalization curves, showing typical Gaussian generalization for less intense stimuli, but flat and high response for more intense stimuli [16].

Little work, however, has utilized olfactory stimuli. In humans, changes in odor concentration of 100-fold or more can be perceived as producing a different odor quality that is as different as a new chemical [25]. Further, research suggests that rodents generalize from a trained odor to concentrations 10-fold lower (or greater) than the trained odorant but show significantly lower responding beyond a 10-fold change in concentration [24,26].

To what degree animals can be trained to generalize or discriminate odor concentrations that are within a perceptually similar range has yet to be tested, to our knowledge. Further, this information would have important implications for the use and capabilities of detection dogs. For example, in the detection dog handler community, it is frequently, but anecdotally, reported that dogs may show great proficiency in detecting a target odor at a concentration they are familiar with, but fail to show interest when the odor is presented at a different concentration.

Another important application is for when dogs are trained in certain detection applications, such as oil spill remediation. Dogs trained to detect oil from leaking pipes or spills are deployed to rapidly identify and screen for potential oil sources that require remediation. However, it may not be uncommon for trace concentrations of oil to appear when remediation may not be feasible, and, thus, dogs need to be trained to ignore such trace, yet detectable, concentrations and respond only to concentrations exceeding some threshold. However, little research has investigated such procedures for training this type of olfactory discrimination, although such learning induced changes to generalization gradients have long been known for visual stimuli [17,27].

The aims of this project were therefore: (1) to assess dogs’ spontaneous generalization to lower concentrations of a trained odorant (i.e., the range of lower concentrations dogs alert to without explicit/additional training to different concentrations); and (2) to evaluate whether training can suppress responding to a concentration to which dogs spontaneously generalized, while maintaining responding to higher concentrations.

## 2. Materials and Methods

Animals. Four mixed breed dogs (see Table 1) were utilized and housed at Texas Tech University Canine Olfaction Lab and were participating in a training program to increase adoptability. Dogs’ were sourced from two local animal shelters and were housed with indoor and outdoor kennels. Dogs’ backgrounds were unknown, but all dogs were presumable naïve to detection training. Dogs received twice daily walks, social enrichment and training. Procedures were reviewed and approved by the Texas Tech University Institutional Animal Care and Use Committee (ACUC# 20010-01).

**Figure 1:**
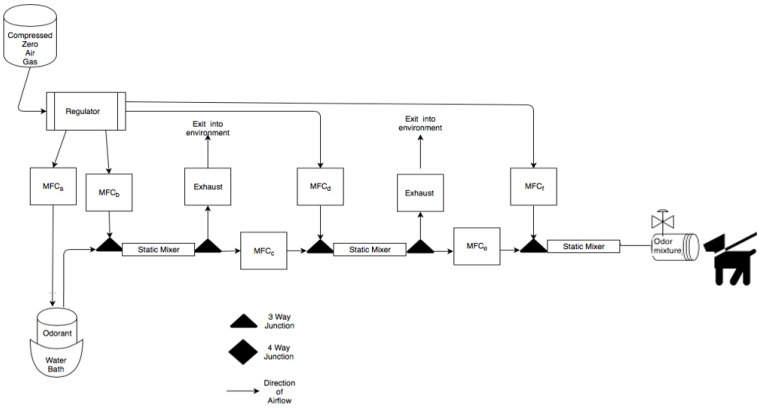
Schematic of the air-dilution olfactometer. Air flow path is shown from left to right and dog is shown evaluating the odor port. Briefly, compressed zero air is used to collect odorant from a sealed glass odorant jar in a water bath. The vapor stream is then systematically diluted using mass air flow controllers and turbulent static mixers. The odor line is then delivered to the odor port.

Air Dilution Olfactometer. To measure thresholds, an air dilution olfactometer that produces one diluted or clean airline was used (see Figure 1). This device takes clean compressed “zero” air and utilizes Alicat^®^ (Tucson, AZ, USA) gas mass air flow controllers to do a serial air dilution of an odorant held in a glass vial designed for volatile organic compound sampling that rested in a temperature-controlled bath at 38 °C. All odor whetted parts are made of Polytetrafluoroethylene (PTFE, Teflon), Stainless Steel 316 and glass per olfactometry standards. A final stimulus flow rate of 10 L/min was used for all odor concentrations and clean air stimuli. The final odor port was constructed of PTFE. The odor port was wiped and cleaned daily with water and ethanol.

Odorant. All dogs were trained to detect isoamyl acetate (CAS #123-92-2). To reduce contamination within the olfactometer, the odorant was pre-diluted in mineral oil to a liquid dilution of 10^−2.5^ *v/v*. The odorant was prepared fresh weekly.

Odorant delivery validation. To validate odorant delivery prior to the experiment, a 10^−2^ *v/v* dilution of isoamyl acetate was used to generate odorant stimuli to be read by a photo-ionization detector (PID, 200b miniPID, Aurora Scientific^®^ (Aurora, ON, Canada). The miniPID was placed in the odor port (medium gain, high pump) where a dog would place its nose. The mean of 200 samples at each of five different concentrations (0.1, 0.07, 0.05, 0.025 and 0.01) was taken during the stable phase of odor activation to create a calibration curve using the mean PID voltage reading with the theoretical vapor dilution generated. The range of vapor dilutions used was higher than the range of concentrations presented to the dog, so that the odorant was sufficiently concentrated to be readable by the PID. Extrapolation to lower concentrations outside the PID reading range was assumed.

Preliminary Training. Dogs were trained on a Go/No-Go task. Dogs were presented with a polytetrafluoroethylene odor port approximately 51 cm from the ground, an exhaust line to remove the odorant, infrared sensors to detect dog presence and a retractable lever that was present during the entire session. An automated cover restricted access to the port between trials, such that the dogs were only able to access the odor port during designated periods. The intertrial interval (ITI) was 20 s and the automated cover prevented access to the odor port during the ITI. An exhaust fan evacuated residual odor during the ITI and the olfactometer initiated the odor (or no odor) presentation for the subsequent trial during the ITI, such that, at the end of the ITI, the stable odor concentration was ready for the next trial.

An automated feeder (SuperFeeder ™, Mount Juliet, TN, USA) rewarded dogs for correct responses. The target odor was presented as a 0.01 air dilution of the diluted isoamyl acetate odorant (10^−2.5^ *v/v*). Dogs “alerted” by holding their nose in the odor port for 3 s, measured by infrared beams, or by pressing the lever. Three dogs tended to make a “nose hold” response, whereas one dog made mostly lever presses. Both responses were counted as “indication” responses and were provided as options to suit the dog’s preference for a more active or passive alert. The “no-go” response was comprised of not holding their nose in the odor port for more than 3 continuous seconds and by not pressing the lever.

Dogs were initially trained to the automated go/no-go device by placing a piece of food in the odor port until the dog learned to insert their nose. Afterwards, shaping to the target odor, isoamyl acetate, with a clicker and food reward was repeated until the dog indicated on the odor in the port. Dogs were taught to “alert” by either holding their nose in the odor port for 3 s or by pressing the lever. The final response was determined simply by which the dog learned first and both responses were allowed. Dogs were trained to press the lever by placing a piece of food on the lever until the dog learned to press with its nose.

Continuous Reinforcement Training. Once dogs learned to operate the olfactometer independently (without experimenter assistance and experimenter outside of the testing area), they were trained in 40 trial sessions to discriminate clean air from a 0.01 air dilution of the odorant, completely under computer control (see Table 2). Correct responses (hits and correct rejections) were reinforced with delivery of a treat from an automated feeder. Misses and false alarms were not reinforced. If dogs made two consecutive incorrect responses of the same type (miss or false alarm) without an intervening correct response, a correction was conducted. Instead of terminating the trial without food for the incorrect response, the olfactometer continued to present the stimulus until the dog made the correct response (made an alert or rejection response). The trial was still scored as incorrect, but these trials were used to maintain motivation if multiple incorrect responses were made. Once dogs achieved 75% accuracy or higher for two consecutive sessions (binomial test, *p* < 0.0001), dogs moved on to intermittent reinforcement training.

Intermittent Reinforcement Training. After successfully reaching criterion with continuous reinforcement, dogs were adjusted to an intermittent schedule of reinforcement in which the probability of reinforcement for any correct response was uniform at 0.8 (see Table 2). The purpose of these sessions was to adjust dogs to expect that not all responses to the target odor, or all no-go responses to the clean stimulus, will be reinforced. This is important for the subsequent generalization testing where non-reinforced probes are inserted as 20% of the trials. Thus, intermittent reinforcement training was used to prepare dogs to not receive feedback (reinforcement) on 20% of trials. Dogs received two consecutive sessions of intermittent reinforcement training to prepare for generalization testing.

Generalization Test 1. Immediately following intermittent reinforcement training, dogs underwent eight 40-trial sessions of generalization testing (see Table 2). Generalization testing was identical to training except that 20% of the trials (8 of the 40 trials) were non-reinforced probe trials to measure spontaneous generalization to different concentrations. No corrections were made for responses on probe trials. On half of the sessions, dogs received two trials of each of the following probe air dilutions of the target odor (isoamyl acetate diluted to 10^−2.5^ *v/v* in mineral oil): 0.003, 0.001, 0.0003 and 0.0001. On the remaining half of sessions, two trials of the following air dilutions were tested: 0.0075, 0.005, 0.0025 and 0.00075. Testing was alternated between the two sets of probe dilutions for all 8 sessions, yielding a total of eight probe trials of each dilution for each dog.

Concentration Discrimination. Following Generalization Test 1, we identified the lowest concentration dogs (as a group) were significantly more likely to alert toward than the clean air stimulus. To evaluate if dogs can learn to accurately discriminate between an odor to which they spontaneously generalize to (0.0025 dilution: see Results) from the trained concentration (0.01), six training sessions (40 trials per session) were conducted in which dogs were required to discriminate these two concentrations. The 0.0025 dilution replaced all clean air trials (0.01 vs. 0.0025) and only rejection responses toward the 0.0025 concentration and alert responses to 0.01 were reinforced (see Table 2).

Generalization Test 2. Following the six concentration training sessions, eight generalization test sessions were conducted identically to Generalization Test 1.

Compound Generalization Test and Discrimination. Following Generalization Test 2, to evaluate if alerting to low concentration odors can be further suppressed, another series of eight generalization test sessions were conducted. However, different from the previous generalization tests, the contingencies in effect for Concentration Discrimination training (0.01 vs. 0.0025) remained in effect for 80% of the trials during the sessions. Alerts to a concentration of 0.0025 were therefore not reinforced and no-go responses to 0.0025 were. Generalization probes to 0.0025 were not conducted (because they were explicitly reinforced) and instead replaced with the concentration of 0 (clean air) to assess responding to clean air (see Table 2).

Statistical Analysis. For early training and concentration training, we calculated accuracy as the number of correct trials (alert to the target stimulus and no alert to the negative stimulus) divided by the total number of trials. To assess generalization on non-reinforced probes, we utilized probability of a response, which refers to the number of trials the dog made an alert to that concentration divided by the total number of trials the concentration was presented. This metric was used for generalization tests because there are no “correct” or “incorrect” responses for the probes. The probability of a response to the 0-concentration stimulus indicates the overall false alarm rate and is displayed in the relevant figures. To analyze the probability of a response during generalization tests statistically, a generalized linear mixed-effect model was used for this binomial data (response or no response) using a logit-link. For all models, Dog ID was used as a random effect. The lme4 package [28] in R (R version 3.5.1, www.r.project.org; R Core Team, Vienna, Austria, 2018) was used to fit models. Post-hoc tests were conducted using the lsmeans package [29].

## 3. Results

**Figure 2:**
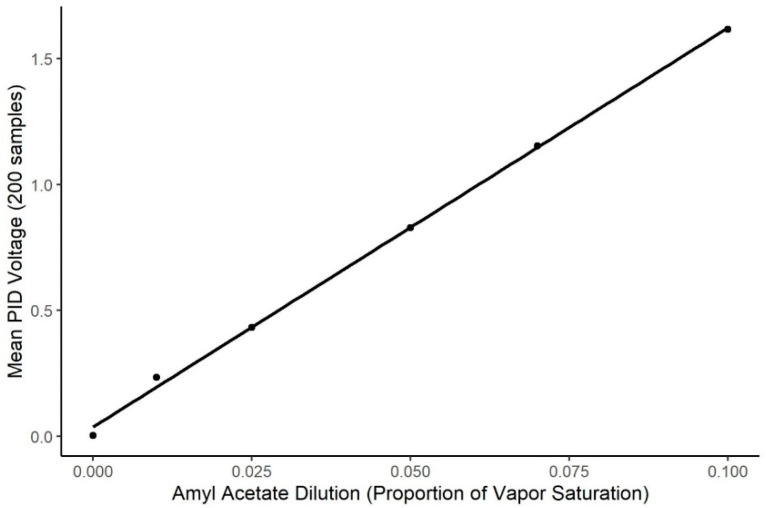
Calibration of Air Dilution Olfactometer. Figure shows the systematic change in the Photo Ionization Detector output as a function of systematic changes in the air dilution olfactometer (R^2^ = 0.998). *X*-axis shows the air dilution in effect on the olfactometer and *Y*-axis shows the change in sensor output.

The air dilution olfactometer calibration curve is shown in Figure 2. The PID output was highly related to systematic changes in the air dilution olfactometer within the dilution range tested (*t* = 804.8, *p* < 0.0001; R^2^ = 0.998). However, the PID showed little detection of the 0.01 concentration, indicating the concentrations used by the dogs for this study were below the detection limits of the PID, and, therefore, accuracy in odor delivery for the concentrations presented to the dogs must be assumed from extrapolation from the data within the sensor’s limits of detection.

**Figure 3:**
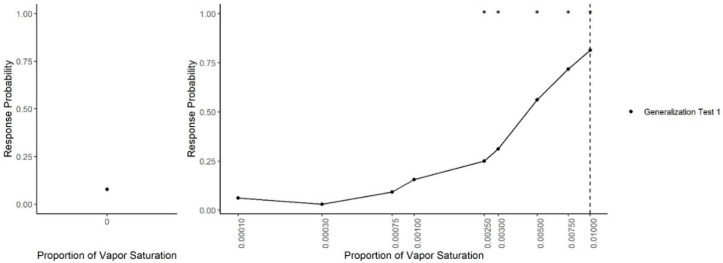
Generalization Test 1. The spontaneous generalization to a range of air dilutions of the trained odorant (0.01; dashed line): (**Left**) the response to a clean air stimulus; and (**Right**) the range of concentrations on a log scale. * indicates the probability of response was greater than the probability of response to the clean stimulus *p* < 0.05.

**Figure 4:**
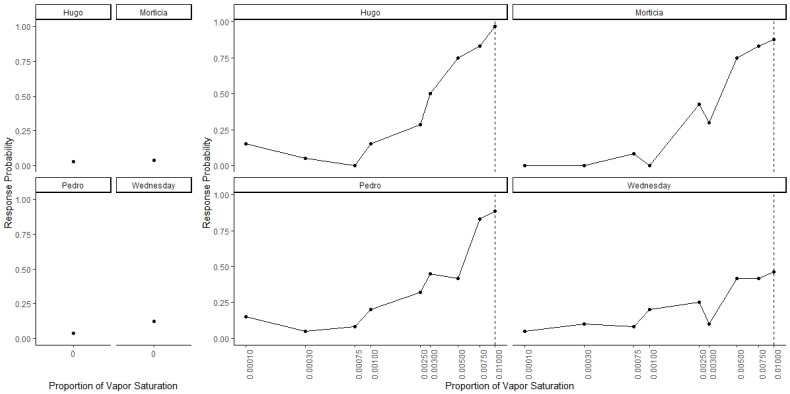
Individual Data for Generalization Test 1. Individual data for all four dogs. Dogs showed similar generalization patterns that reflect the group trends.

Figure 3 shows dogs’ spontaneous generalization (proportion of trials dogs made a response to that concentration) to a 100-fold range of concentrations below the trained target concentration. The left panel shows the response to the clean air stimulus (0 concentration), which reflects the overall false alert rate. A generalized-linear mixed-effect model was used to compare the probability of an alert at each probed dilution in comparison to the clean air (negative) stimulus. Overall, there was a significant effect of odor concentration on dogs’ probability of making an alert response (X^2^ = 750, df = 9, *p* < 0.001). Dunnet-adjusted post-hoc tests, comparing the probability of response at each concentration to clean air, indicate that dogs were significantly more likely to alert to the trained odorant at a 0.01 dilution (z = 19.58, *p* < 0.001), the probe dilution 0.0075 (z = 8.07, *p* < 0.001), probe dilution 0.005 (z = 6.97, *p* < 0.001), probe dilution 0.003 (z = 4.01, *p* < 0.001) and probe dilution 0.0025 (z = 3.13, *p* = 0.01). Dogs, however, alerted to the remainder lower concentrations statistically at the same rate as clean air (0.001, 0.00075, 10^−3.5^ and 10^−4^, all *p* > 0.05). Thus, dogs spontaneously alerted to any concentration within a 10-fold dilution of the target odor but failed to respond to the 10-fold dilution itself and any dilution lower. Figure 4 shows the individual dog generalization curves. All dogs showed similar patterns of responding to that of the group trend, although Wednesday showed an overall lower probability of responding across all concentrations.

Overall, Generalization Test 1 indicated that a concentration of 0.0025 was the lowest concentration dogs spontaneously alerted towards greater than clean air. To evaluate whether dogs could be trained to discriminate between concentrations, Concentration Discrimination training was initiated in which alerts to 0.01 dilution were still reinforced but alerts to 0.0025 were not (but no-go responses to 0.0025 were reinforced). Only the 0.01 and 0.0025 stimuli were presented during the six concentration training sessions. This training would reveal if dogs could successfully learn this discrimination between concentrations.

**Figure 5:**
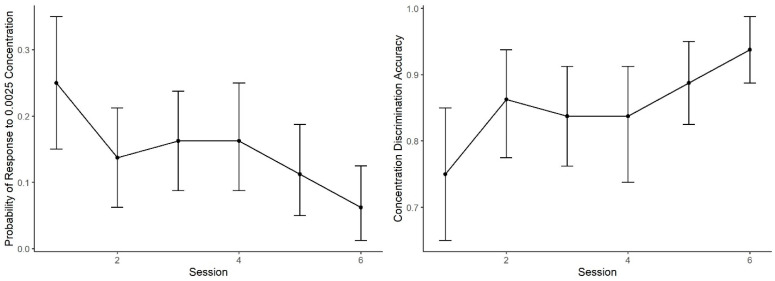
Concentration Discrimination Training. The mean performance and 95% confidence intervals during the concentration training sessions: (**Left**) the probability dogs alerted to the 0.0025 concentration, which decreased across sessions; and (**Right**) the overall session accuracy, where alerting to 0.01 was correct and not alerting to 0.0025 was correct.

Figure 5 shows the mean probability of alerting to the 0.0025 concentration (proportion of trials in which dogs made an alert). During Session 1, the probability of an alert to 0.0025 was nearly identical to the probability in Generalization Test 1 (Figure 3). As training proceeded, however, the probability of response to this concentration subsequently decreased. A generalized linear mixed effect model predicting alert probability to the 0.0025 concentration across the six sessions indicates dogs showed lowering probability in alerting to the 0.0025 concentration across sessions (effect of day: z = 3.00, *p* < 0.001). In addition, accuracy (i.e., mean number of correct responses by alerting to 0.01 and not alerting to 0.0025, See Figure 5) showed improvement with training days, reaching over 90% accuracy. The effect of day, however, did not reach statistical significance (z = 1.07, *p* = 0.28).

**Figure 6:**
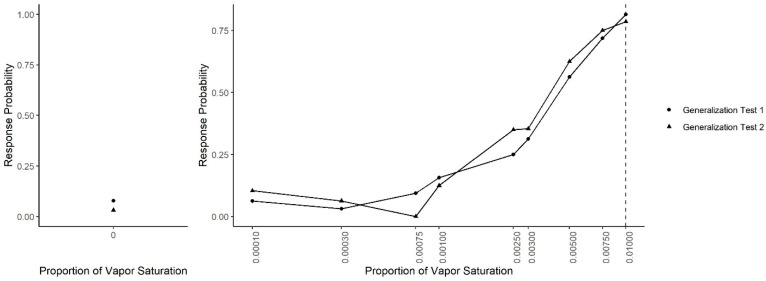
Combined generalization testing. The spontaneous generalization to a range of air dilutions of the trained odorant (0.01; dashed line) for Generalization Test 1 and Generalization Test 2: (**Left**) the response to a clean air stimulus; and (**Right**) the range of concentrations on a log scale.

Following Concentration Discrimination, the generalization test was repeated (Generalization Test 2; see Figure 6). A generalized linear mixed-effect model, predicting response probability as a function of odor concentration, the test number (Generalization Test 1 or Test 2) and their interaction, indicated that there was not a significant interaction (X^2^ = 13.92, df = 9, *p* = 0.13), suggesting that responding to each concentration did not differ between the two generalization tests. However, to break down comparison between the two generalization tests at each concentration, Tukey-adjusted post-hoc tests comparing the probability of an alert between Generalization Tests 1 and 2 at each concentration were run. Post-hoc tests indicated that, in Test 2, dogs were significantly less likely to false alert on the clean air compared to Test 1 (z = 3.06, *p* = 0.002). The probability to alert to all other concentrations, however, was not significantly different between Tests 1 and 2, including the 0.0025 concentration (z = 1.053, *p* = 0.29) in which dogs were successfully taught to not alert toward in the immediately previous Concentration Discrimination sessions.

Following Generalization Test 2, we evaluated whether generalization might be relative to the contingencies in effect during the sessions (i.e., is a clean air stimulus the no-go stimulus or another concentration). Dogs’ generalization was next evaluated in identical generalization sessions except that 0.0025 was used as the no-go stimulus, thereby combining contingencies of the Concentration Discrimination and Generalization Test sessions.

**Figure 7:**
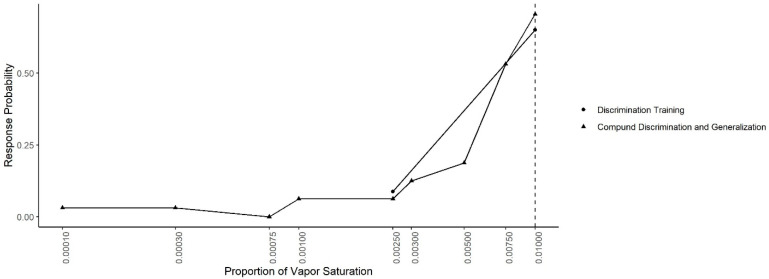
Concentration Discrimination and Compound Generalization. The response probability for the last two sessions of the discrimination training phase (0.01 vs. 0.0025) and the Compound Discrimination and Generalization test. Dashed line shows the target odor concentration.

Figure 7 shows the mean probability of response to each concentration during the Compound Generalization and Discrimination and during the last two sessions of the Concentration Discrimination training. In both conditions, the same contingency was in effect (alerts to 0.01 reinforced and alerts to 0.0025 not reinforced). A generalized-linear mixed effect model, predicting response probability as a function of odor concentration, session type and their interaction, indicated there was no significant interaction between the odor concentration and session (X^2^ = 2.77, df = 1, *p* = 0.10) or main effect of session (X^2^ = 0.68, df = 1, *p* = 0.40), indicating responding was similar in both conditions.

**Figure 8:**
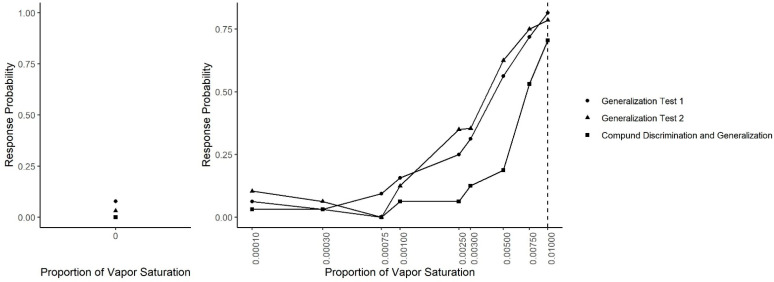
Generalization across all tests. The spontaneous generalization to a range of air dilutions of the trained odorant (0.01; dashed line) for Generalization Test 1, Generalization Test 2 and Compound Discrimination and Generalization: (**Left**) the response to a clean air stimulus; and (**Right**) the range of concentrations on a log scale.

Figure 8 shows the results across all generalization sessions (Generalization Test 1, Generalization Test 2 and Compound Generalization and Discrimination). A generalized linear mixed-effect model was fit in which the probability of an alert was predicted by odor concentration, the session type (Generalization Test 1, Generalization Test 2 or Compound Generalization and Discrimination) and their interaction. There was a significant interaction between the session type and odor concentration (X^2^ = 44.45, df = 18, *p* = 0.001). Tukey-adjusted pairwise post-hoc tests at each concentration indicated that at the target odor concentration (0.01), the probability of an alert was lower in the Compound Discrimination and Generalization phase than in the Generalization Test 2 phase (z = 3.17, *p* = 0.004) and the Generalization Test 1 phase (z = 4.38, *p* < 0.001). At the next lower concentration (0.0075), there were no statistically significant differences between the three phases. At a concentration of 0.005, the probability of an alert was lower in the Compound Discrimination and Generalization sessions than the Generalization Test 2 (z = 3.03, *p* < 0.001) and the Generalization Test 1 (z = 3.03, *p* = 0.007). At the next lower concentration (0.003), a similar result held, such that the probability of an alert tended to be lower in the Compound Discrimination and Generalization sessions than Generalization Test 1 (z = 1.82, *p* = 0.16), and it was almost significantly lower than in Generalization Test 2 (z = 2.28, *p* = 0.06). At the concentration used for training dogs not to alert (0.0025), dogs showed significantly lower probability to respond in the Compound Discrimination and Generalization session compared to Generalization Test 1 (z = 3.62, *p* < 0.001) and Generalization Test 2 (z = 7.13, *p* < 0.0001), highlighting successful reduction in responding to this concentration. For all the remaining lower probed concentrations, post-hoc tests revealed no significant differences between the three sessions types, except for the clean air (no odor) concentration. For the clean air stimulus, the lowest probability to alert (i.e., false alert) was in the Compound Discrimination and Generalization condition, but this was not significantly different from Generalization Test 1 (z = 0.02, *p* = 0.99) and Generalization Test 2 (z = 0.02, *p* = 0.99). As previously shown, however, the probability of a response to clean air was lower in Generalization Test 2 than in Generalization Test 1 (z = 3.06, *p* = 0.006).

## 4. Discussion

The results indicate that dogs spontaneously generalized in non-reinforced probes within a 10-fold range of the trained odor concentration. At a 10-fold dilution and lower, the probability of response was no greater than it was to clean air. This is an important finding as it suggests that dogs only spontaneously generalize to a limited range of an odor concentration without explicit training. Importantly, however, dogs were not reinforced for responding to lower concentrations, therefore it is expected that generalization would be greater if dogs had reinforced training for lower concentrations.

One possible explanation for the minimal concentration generalization is that perhaps dogs could simply not detect a concentration <10-fold dilution of the target odor. However, assuming a vapor pressure of ~6 mmHg (PubChem, Isoamyl acetate, CID = 31,276), the anticipated vapor concentration (assuming ideal conditions for Raoult’s law) would be approximately 90 parts-per-billion for the 10-fold dilution of the target odorant. Given that recent work has shown amyl acetate thresholds to be close to 1 parts-per-trillion for dogs [30,31], our test concentrations should be well above the dog detection limits. This suggests that the low probability of response was a generalization failure, not a sensitivity failure. We selected the concentration range used in this study to optimize dog detection (sufficiently salience) without being too concentrated to produce nuisance contamination in the system. Future work, however, can evaluate whether the same results are observed at varying starting concentrations for the trained odorant.

Results from the Concentration Discrimination sessions indicate dogs can readily learn to discriminate a 0.0025 dilution from a 0.01 dilution (a difference of only 0.0075), achieving greater than 90% accuracy within six sessions. Dogs successfully made go responses to the 0.01 dilution, and no-go responses to the 0.0025 dilution. Interestingly, however, despite successfully making nearly all no-go responses to the 0.0025 concentration, when the second generalization test was conducted, responding at the 0.0025 concentration immediately rebounded. This suggests that the training context (i.e., the dilutions used within a session and their contingencies) may have an important impact on generalization responding.

By inserting probe trials within a session in which 0.01 is discriminated from 0, dogs may be more likely to respond to any stimulus smelling close to the 0.01 target. In other sensory domains, generalization has been demonstrated to be under relative stimulus control vs. absolute control of the stimulus. For example, the early generalization gradient work demonstrated that when an extinction stimulus is introduced (i.e., a stimulus to which alerts are not reinforced), responding is shifted away from the extinction (non-reinforced) stimulus, leading maximum responding to values exceeding the trained stimulus, termed a “peak shift” (i.e., not the exact value of the reinforced stimulus [27,32,33]). It appears a similar process may occur with respect to olfactory stimuli.

In the final session, in which probe trials were inserted within concentration training (0.01 dilution was discriminated from the 0.0025 concentration), we found that no-go responses to the 0.0025 concentration were maintained (i.e., alerts suppressed). In addition, responding to the 0.01 dilution was maintained, although the probability of a response was lower than observed in the other generalization tests. This could suggest that had we tested generalization to higher concentrations, we may have observed a peak shift away from the 0.0025 stimulus for higher concentrations of the target odorant.

Importantly, however, this does suggest that dogs can be trained to alert to concentrations greater than some threshold and not respond to lower concentrations that the dog perceives as being similar to the target concentration. These results further suggest that successful application of this may require constant training with the low concentration odorant as a no-go stimulus to maintain suppression of responding and prevent alerting to the low concentration from rebounding, as it did in the second generalization test.

Another interesting finding is that false alarms decreased after Concentration Discrimination training. This may result from training dogs not to respond to the low, but detectable concentration odor (0.0025), which made the clean air stimulus even more perceptible as a no-go stimulus. However, because this was not experimentally tested, it is possible that simply additional training led to this improvement.

There are several important limitations to the present findings. The first is the required inherent training order in the sessions. This makes it challenging to determine if the decrease in false alarms may simply be a maturation/experience effect across time or may reflect behavioral changes due to training the 0.0025 concentration as a no-go stimulus. Ideally, we would have further alternated between generalization test sessions and the compound discrimination and generalization sessions to assess steady state responding, reducing experience effects. However, the 2020 SARS-CoV-2 pandemic prevented further testing between the conditions, but this would remain an important follow-up.

Another limitation is that we explicitly reinforced correct no-go responses. This differs from typical peak-shift protocols that suppress responding with extinction (i.e., simply not reinforcing any response except an alert). We opted not to use this option due to concern that non-reinforced probe trials would thin the reinforcement schedule too much to maintain responding. We do not expect this would have changed the direction of any effects observed but may have more effectively suppressed alerting to the low concentration odorant, given the additional reinforcer to not respond in addition to extinction of any alerts.

The present results also have important implications for detection dogs. It suggests that dogs can be trained to actively not respond to concentrations of a trained odorant below a desired threshold. This could be useful for oil-leak detection dogs, in which detection of low concentrations of oil may not present a remediation issue. However, the results suggest that continuous training to not alert to low concentrations may be necessary. Another implication of these results suggests that dogs do not spontaneously generalize to a wide range of concentrations lower than the concentration of the odorant trained. This suggests that, if low concentrations are necessary to detect, explicit training at those concentrations will be important.

Another important implication for detection dog training is regarding potential training problems. If odor samples are not prepared carefully, and samples that a trainer may think are “clean” are incidentally contaminated with a significant amount of the odorant, this research suggests dogs can learn to not alert to the contaminated container and still respond to the higher concentration target. From the trainer’s perspective, the dog may seem proficient in detection. Critically, however, the dog may explicitly ignore significant concentrations of the odorant that resemble the contamination concentration, which may only be slightly lower than the trained odor concentration (the present study indicates dogs can successfully ignore a 25% dilution of the trained odor). This may lead to poorer than expected performance in operational scenarios.

Last, it is important to highlight the potential impact detection training itself may have on the welfare of the dogs. Recent research has suggested that scent training can induce a positive judgement bias in dogs [34]. Given that the population of dogs participating in this study were in a training to adopt program, training and procedures that improve the welfare of animals is an important consideration. Future research should evaluate more explicitly what, if any, impact the detection training program has on the overall welfare of the dogs.

## 5. Conclusions

In conclusion, the present results suggest dogs may only spontaneously generalize to concentrations less than a 10-fold dilution of the training odorant. Further, dogs can learn to successfully discriminate between a higher concentration odorant and a 25% dilution of that odorant within six training sessions. Lastly, dogs can successfully learn to only alert to concentrations greater than a certain threshold, but this may need to be done within a context of consistent training to not respond to the lower concentration.

## Figures and Tables

**Table 1:** Description of dogs.

Dog ID	Approximate Age (Years)	Approximate Weight (kg)	Visual Breed Estimate	Reproductive Status
Wednesday	1	19.00	Labrador Retriever Mix	Spayed Female
Morticia	1	22.65	Labrador Retriever Mix	Spayed Female
Pedro	2	25.50	Pit Mix	Neutered Male
Hugo	2	22.85	Pit Mix	Neutered Male

**Table 2:** Training sessions conducted. The target stimulus (alerts reinforced) and non-target stimulus (no-go response reinforced) of the trials are shown. The training is shown in the order in which it was conducted (top to bottom).

Training Phase (In Order)	Target Odor Dilution (S^+^)	No-Response Stimulus Dilution (S^−^)	Probes?
Continuous Reinf	0.01	0.00	No
Intermittent Reinf	0.01	0.00	No
Generalization Test 1	0.01	0.00	Yes
Concentration Discrimination	0.01	0.0025	No
Generalization Test 2	0.01	0.00	Yes
Compound Generalization Test and Discrimination	0.01	0.0025	Yes

## Data Availability

All data are provided as Supplementary Materials to this article.

## Supplementary Materials

The following are available online at https://www.mdpi.com/2076-2615/11/2/326/s1, File S1: Data description, File S2: Raw Data.
